# Pediatric Facial Lacerations: Evidence-Based Over-the-Counter Wound Care and Scar Prevention for Clinicians and Caregivers

**DOI:** 10.7759/cureus.85909

**Published:** 2025-06-13

**Authors:** Kerolos Antonious

**Affiliations:** 1 Dermatology, Rutgers Robert Wood Johnson Medical School, Piscataway, USA

**Keywords:** absorbable sutures, caregiver education, let anesthetic gel, over-the-counter wound care, pediatric facial lacerations, scar mitigation strategies, silicone-based therapies, tissue adhesive closure, wound healing in children

## Abstract

Pediatric facial lacerations are common injuries that carry important cosmetic and psychosocial implications. Prompt, evidence-based management focusing on optimal wound closure, early wound care, and scar prevention is essential to achieving favorable long-term outcomes. This review summarizes findings from a comprehensive literature review conducted from 2010 to 2025, drawing from randomized controlled trials (RCTs), meta-analyses, systematic reviews, and clinical guidelines addressing pediatric facial laceration repair, wound care practices, scar prevention, and caregiver involvement. Practical interventions accessible over the counter (OTC) were emphasized. Evidence supports the use of topical anesthetics such as lidocaine-epinephrine-tetracaine (LET) gel to reduce procedural pain, copious irrigation with potable water, and wound closure methods tailored to the injury’s characteristics, including tissue adhesives and absorbable sutures. Routine systemic antibiotics are not indicated for clean facial wounds. Early moist wound care with petrolatum promotes faster healing, while scar prevention measures such as silicone therapy, sun protection, scar massage, and tension offloading techniques significantly improve cosmetic outcomes. Over-the-counter products such as onion extract and vitamin E offer minimal benefit compared to silicone-based therapies. Parental education on wound care, emotional reassurance, and scar management plays a pivotal role in optimizing healing and minimizing the psychosocial impact of facial scars in children. Through collaboration between clinicians and caregivers, excellent functional and aesthetic outcomes can be achieved, helping children heal both physically and emotionally.

## Introduction and background

Pediatric lacerations are a frequent cause of emergency visits. In the United States alone, over six million lacerations are treated annually in emergency departments [1]. Facial lacerations are particularly common in young children due to falls and play injuries, and even relatively minor facial cuts can cause significant distress for patients and families. Beyond the immediate concern of wound closure, the long-term cosmetic outcome is paramount. Children will live with their scars as they grow, and visible facial scars can affect self-esteem and social development [2]. Notably, scars in pediatric patients may enlarge or become more noticeable over time as the child’s face grows, underscoring the importance of optimal initial management [3]. Therefore, clinicians must prioritize techniques that promote wound healing while minimizing infection and scarring.

Scar formation is an inevitable consequence of full-thickness skin injury; however, its extent can be modulated by evidence-based care [3]. In recent years, high-quality studies and updated clinical guidelines have advanced our understanding of best practices in laceration repair and scar prevention. This review discusses the acute management of pediatric facial lacerations, including wound preparation, anesthesia, and closure, followed by post-closure wound care and proven strategies for scar minimization. We emphasize interventions that are readily available (over-the-counter (OTC) or easily accessible) and practical for both clinicians and caregivers. By integrating current evidence from randomized controlled trials (RCTs), meta-analyses, and expert consensus, we aim to provide a clear guide to managing pediatric facial lacerations in a manner that optimizes cosmetic outcomes and empowers caregivers in scar prevention. The recommendations align with contemporary pediatric wound care principles and emphasize high-quality evidence from 2020 onward.

## Review

Initial evaluation and wound preparation

Proper initial management of a pediatric facial laceration lays the foundation for successful healing and minimal scarring. The clinician should begin with a thorough evaluation of the wound. Key factors include the mechanism of injury, wound age, degree of contamination, and involvement of any critical structures (e.g., eyelid margin and vermilion border of the lip). Most simple facial lacerations in children are due to blunt trauma (falls or collisions) and are relatively clean; however, animal bite wounds or wounds with foreign debris require special attention as discussed later [4]. Ensuring the child’s overall stability and addressing pain and anxiety are top priorities before any wound manipulation.

Pain control and anxiety management

Adequate analgesia and child-friendly approaches significantly improve the experience and outcome of laceration repair. Topical anesthesia is an evidence-based first step in children. A lidocaine-epinephrine-tetracaine (LET) gel applied to the laceration for approximately 20-30 minutes can provide effective local anesthesia to small facial wounds without the need for injections. In a prospective study, LET gel pretreatment was significantly less painful than infiltrated anesthesia (after eutectic mixture of local anesthetics (EMLA) cream) in children while providing equivalent pain control during the laceration repair [5]. This approach eliminates the need for immediate needle infiltration in many cases and is now considered a superior initial anesthetic method for pediatric lacerations in the emergency setting. If the wound is too large or deep for topical anesthesia alone, buffered lidocaine can be injected locally after LET has partially numbed the area. Techniques to minimize injection pain, such as using small-gauge needles, injecting slowly, and introducing the needle through the wound edges rather than intact skin, should be employed [6]. Adjunct measures such as distraction techniques, guided imagery, or the presence of a child life specialist can further alleviate a child’s anxiety during the procedure.

In certain situations, especially with very young or fearful children and complex lacerations, sedation may be necessary to accomplish a safe repair. Traditionally, options include intranasal midazolam for mild sedation or ketamine (administered intramuscularly or intravenously) for dissociative analgesia when deeper sedation is required [7]. However, sedation carries inherent risks and prolongs recovery, so it should be reserved for cases where less invasive methods are insufficient. The growing use of tissue adhesives (skin glues) has reduced the frequency of sedation or general anesthesia for laceration repair, as these methods are faster and less painful than suturing for appropriate wounds [8].

Wound cleansing

Once adequate analgesia is achieved, the wound must be cleaned meticulously to prevent infection and optimize healing. Gentle irrigation with a copious volume of solution is the most important step in removing bacteria and debris. Current evidence indicates that sterile saline is not mandatory; clean potable tap water is equally effective for the irrigation of uncomplicated wounds [9]. A Cochrane review and multiple randomized controlled trials have shown no increase in infection rates when wounds were irrigated with tap water compared to sterile saline [9]. Therefore, caregivers at home can be advised to rinse a fresh cut with clean running water if immediate medical care is not available, as an important first aid measure.

The irrigation pressure should be sufficient to dislodge contaminants but not so high as to cause tissue damage. Typically, using a syringe and splash guard (if available) to direct saline with moderate pressure is effective. For facial lacerations, aggressive scrubbing is usually unnecessary and discouraged, as facial skin is well vascularized and tends to be clean. Scrubbing can also bruise delicate tissues. After irrigation, any visible foreign material can be carefully removed with sterile forceps. Antiseptic solutions such as povidone-iodine or chlorhexidine are often used to prepare the surrounding skin, but should not be poured directly into the wound bed. Studies have found that agents such as hydrogen peroxide or strong iodine solutions can be cytotoxic to healthy tissue and may impair wound healing [10]. For example, hydrogen peroxide, while useful for loosening dried blood, is detrimental to wound healing if used repeatedly because it can kill fibroblasts and keratinocytes essential for tissue repair [10]. Thus, the wound should primarily be irrigated with water or saline, and any cleansing with antiseptics should be limited to gentle dabbing along the wound edges if necessary.

Conservative wound debridement is advised for facial lacerations. Because the face has a rich blood supply, tissue that might initially appear devitalized often recovers, and unnecessary removal of tissue (especially subcutaneous fat or skin edges) can create a larger defect and a more pronounced scar. Minimal trimming of clearly nonviable tissue is sufficient in most cases. Importantly, debridement should avoid indiscriminate removal of subcutaneous fat, as excising fat pads can lead to contour depressions in the healed scar. Gentle handling of tissues and avoidance of crushing or desiccation of wound edges are basic surgical principles that directly correlate with better cosmetic outcomes. The wound edges should also be evaluated for alignment considerations. For example, if an eyebrow is involved, eyebrow hairs should not be shaved, as they are important landmarks for proper alignment and may not fully regrow if removed. Similarly, if the laceration involves a border such as the vermilion border of the lip or the eyelid margin, special care must be taken to align these structures precisely, often requiring layered closure or specialist referral, such as to a plastic surgeon, to achieve optimal results [11].

Wound closure techniques

Selecting the appropriate wound closure method is critical for both functional healing and cosmetic outcome. Pediatric facial lacerations can often be closed with less invasive methods than traditional suturing, thanks to advances in tissue adhesive technology and the generally favorable healing of facial wounds. The choice among sutures, tissue adhesive (glue), or adhesive strips (Steri-Strips®) depends on the laceration’s characteristics (size, depth, configuration, and tension) and the child’s cooperation level.

Sutures

Suturing remains the standard for lacerations under tension or those requiring precise alignment (e.g., across the vermilion border or involving deep structures) [12]. On the face, fine sutures (5-0 or 6-0 nylon or polypropylene) are typically used to achieve precise approximation with minimal scarring. If the wound is deep, a layered closure can be done with absorbable dermal sutures (e.g., 5-0 polyglactin) to eliminate dead space, but one must balance this with the added tissue reactivity of some absorbable materials [13]. Interestingly, evidence suggests that routine deep (dermal) sutures are not always needed in facial lacerations that are not gaping; a single-layer closure often suffices and yields similar cosmetic results. The key is to achieve everted wound edges and minimal tension. Techniques such as fine-gauge interrupted sutures or small Steri-Strips® can be used to gently evert edges to prevent a sunken scar, as inverted edges tend to heal with a visible depression. Mattress suture techniques (vertical or horizontal) can help with eversion in select areas but should be used judiciously on the face to avoid suture marks [14].

In pediatric patients, there is a strong preference to avoid the need for suture removal when possible. Randomized trials have shown that absorbable sutures, such as fast-absorbing plain gut, are a viable alternative to nylon for pediatric facial wounds, with no significant difference in long-term cosmetic outcomes or complication rates. In one randomized controlled trial involving 47 children, lacerations repaired with fast-absorbing catgut had equivalent cosmetic appearance at three months compared to those repaired with traditional nylon sutures, and infection and dehiscence rates were similarly low in both groups. Parental satisfaction was high, and importantly, absorbable sutures spared the child a potentially traumatic suture removal visit [15]. Based on such evidence, many clinicians now use absorbable sutures for facial lacerations in young children.

If non-absorbable sutures are placed, they should be removed early. The face heals quickly, and removing sutures in approximately five days (no more than five to seven days) is recommended to minimize track marks on the skin. In some cases, suture removal in as few as three days can be considered for low-tension facial wounds in very young children, as long as the wound is holding well. Adhesive strips can then be applied afterward to support wound healing [16].

Tissue Adhesives

The use of cyanoacrylate tissue adhesives (e.g., octyl cyanoacrylate, marketed as Dermabond®) has transformed pediatric laceration care. These medical “glues” polymerize to bond wound edges together, obviating the need for sutures in many cases. According to a Cochrane review and subsequent studies, tissue adhesives are an acceptable alternative to sutures for small, superficial lacerations in children, with the advantage of significantly reduced procedure time and pain [17]. A recent large prospective study compared long-term outcomes of 96 pediatric facial lacerations closed with tissue adhesive versus 134 with sutures. Blinded observers rated scar appearance at 6-12 months slightly better on average in the adhesive group, and caregivers/patients reported no difference in satisfaction. While early wound dehiscence was more common with glue (approximately 12.5% of cases versus 3%-4% with sutures), most of these were minor and could be managed with secondary closure or Steri-Strips®. Ultimately, both methods produced low infection rates (~1% or less) and favorable cosmetic results in the vast majority of cases. Tissue adhesives also proved more cost-effective and much faster to apply [2].

The indications for using a tissue adhesive in children’s facial wounds include lacerations that are clean, straight, and under low to moderate tension (wounds that would easily come together with gentle pressure) and up to about 3 cm in length. Wounds should not involve mucosal surfaces or areas that are constantly moist, as cyanoacrylate adhesives will not hold if continuously wet with saliva or tears [17,18]. In practice, many forehead, chin, and cheek lacerations in children meet these criteria. The child should ideally be cooperative enough to hold relatively still for the 30-60 seconds needed for the adhesive to set. If the wound edges are not perfectly apposed, a quick Steri-Strip® can be placed to push them together before applying glue.

When using tissue adhesive, the clinician must ensure the wound is thoroughly hemostatic and dry (excess bleeding or moisture will interfere with bonding). The adhesive is applied as a thin layer bridging the wound edges; typically, three layers are applied, allowing 30 seconds between applications. Care must be taken to avoid getting glue into the wound (between the edges) or dripping into unwanted areas (e.g., eyes), as it can cause foreign body reactions if trapped inside the wound or glue skin unintentionally [18].

Adhesive Strips

Adhesive wound closure strips (Steri-Strips®) can be used alone for very superficial lacerations or in conjunction with suture removal. They are essentially thin pieces of porous tape that can hold wound edges together under low tension. A randomized trial in 2002 comparing Steri-Strips® to tissue glue in children found both methods had similar cosmetic outcomes and low complication rates, with strips possibly having a slightly lower risk of dehiscence in areas of movement. However, adhesive strips are somewhat technique-sensitive: the surrounding skin must be dry and free of oil for optimal adhesion, and young children may peel them off if unsupervised [19]. Strips can also be used after suture removal to provide additional support to the wound for a few days.

Staples

Metallic skin staples are generally avoided in facial lacerations because of cosmetic considerations, as they tend to produce more pronounced puncture scars and are less precise in everting delicate facial skin. Staples are more commonly reserved for scalp lacerations or other areas where the cosmetic outcome is less critical. In rare urgent scenarios involving the face, such as a long laceration in a combative child where speed is essential and tissue adhesive is not suitable, a few staples may be used as a temporizing measure but should be removed within a few days and, if necessary, replaced with a more cosmetic closure [20]. A summary of wound closure methods for pediatric facial lacerations is presented in Table 1.

**Table 1:** Wound Closure Methods for Pediatric Facial Lacerations

Method	Typical use case	Advantages	Disadvantages	Citation
Sutures (5-0/6-0)	Moderate-/high-tension facial lacerations; irregular or gaping wounds; critical border repairs (lip and eyelid)	Precise approximation; secure closure even under tension; adjust eversion precisely	Requires anesthesia (local injection); time-consuming procedure; needs removal (unless absorbable)	Tanaydin et al. (2016) [13]
Absorbable sutures	Same indications; young children or uncertain follow-up	No removal needed; proven cosmetic equivalence to nylon	Slightly less tensile strength; potential inflammatory reaction	Luck et al. (2008) [15]
Tissue adhesive (glue)	Straight, clean lacerations ≤2-3 cm under low tension; cooperative child	No needles needed; fast; painless; no removal needed; excellent cosmetic results	Not for high-tension or wet/mucosal areas; early dehiscence risk (~12%)	Fontana et al. (2021) [2]; Ste-Marie-Lestage et al. (2019) [17]
Adhesive strips	Very small, low-tension wounds; adjunct after suture removal or to reinforce glue	Painless; easy to apply; avoids foreign body response	Weak adhesion if wet or oily; the child may remove prematurely	Atkinson et al. (2005) [14]
Staples	Rarely used for facial lacerations; scalp wounds or urgent large wounds elsewhere	Extremely rapid closure; secure hold	Poor cosmetic outcome on the face; prominent staple marks; needs removal	Al-Mubarak and Al-Haddab (2013) [20]

Prophylactic antibiotics and tetanus considerations

One important aspect of laceration management is the prevention of infection. Fortunately, the face has an excellent blood supply, and infection rates for well-managed facial lacerations are very low, typically reported in the range of 1%-5%. Factors that increase infection risk include heavy contamination, puncture wounds, wounds greater than 5 cm, and wounds located in areas with poorer circulation, such as the legs [21]. In contrast, facial wounds tend to resist infection.

Given the low baseline infection rates, routine use of systemic prophylactic antibiotics for pediatric facial lacerations is not indicated in most cases. Randomized trials and meta-analyses have failed to show a benefit of prophylactic oral antibiotics for simple traumatic lacerations in reducing infection incidence. Current practice is to reserve antibiotics for wounds with specific high-risk features, including bites (human or animal), visibly contaminated wounds (e.g., containing dirt or gravel), wounds in immunocompromised patients, and possibly wounds in areas of poor perfusion [22].

Animal Bites

Animal bites on kids’ faces are common and require specific care. In 111 facial bites, 94.5% were caused by dogs, and children under 10 were more likely to be bitten than adults. The most affected (40.5%) was the perioral area. In 74.8% of instances, primary wound closure had the lowest infection rate (4.8%) of all surgical procedures. This supports the recommendation that quick primary closure be the first treatment for facial animal bites for optimum cosmetic and infection effects [23].

Patients received antibiotic prophylaxis in 91.9% of cases, with amoxicillin-clavulanate being the most common (62.1%). Although not statistically significant, patients who did not get antibiotics had a greater infection rate (22.2%) than those who did (6.8%). Therefore, preventative antibiotics, especially amoxicillin-clavulanate, are advised for Lackmann class II or higher wounds or deep tissue involvement [23].

If the animal’s vaccination status is uncertain or the species is at high risk, consider rabies prophylaxis for any bite wound. The study delivered fox-bitten people post-exposure rabies injections, per national standards. Although terrestrial rabies is rare in some countries (like Germany), prophylaxis should be administered if exposure is possible [23].

Clean Traumatic Lacerations

Aside from bite wounds, most clean lacerations do not require antibiotics. This includes wounds repaired with sutures or tissue adhesive in the emergency department, as studies have shown that adding prophylactic oral antibiotics does not significantly reduce infection rates in these scenarios [22]. Overuse of antibiotics can contribute to resistance and exposes the child to potential side effects, such as diarrhea or allergic reactions, unnecessarily. Caregivers can be reassured that meticulous wound cleansing and proper home care, including keeping the wound clean and covered, are usually sufficient to prevent infection in facial lacerations.

Topical Antibiotic Ointments

Topical antibiotic ointments are commonly used by clinicians and advised to caregivers for wound care. The classic teaching was that topical antibiotics (such as bacitracin or polymyxin-containing ointments) might reduce superficial infection and keep the wound moist. However, evidence indicates that a simple petrolatum (Vaseline®) ointment is just as effective as antibiotic-based ointment in promoting healing and preventing infection in clean wounds [18]. A landmark randomized trial in dermatologic wounds demonstrated no difference in healing or infection rates between wounds treated with petrolatum alone versus those treated with bacitracin/polymyxin ointment [18].

The only notable difference was that antibiotic ointments caused a higher rate of local allergic reactions (contact dermatitis), particularly from neomycin and bacitracin. Pediatric skin can be especially sensitive; cases of allergic contact dermatitis from neomycin on a healing wound are not rare [24]. For this reason, many experts now favor plain petroleum jelly to maintain moisture in the wound over antibiotic ointments, unless the wound was grossly contaminated or the patient has a specific risk factor for infection [18,24].

Some guidelines still suggest that a light application of antibiotic ointment is acceptable in the first few days for the prevention of superficial infection. If antibiotic ointment is used, mupirocin is a good choice for facial wounds as it has a low sensitization rate and covers skin flora, including* Staphylococcus aureus*. In any case, keeping the wound moist (with petrolatum or ointment) and covered is more important than the presence of an antibiotic in the ointment for preventing infection and promoting optimal healing [18].

Tetanus Prophylaxis

Every pediatric wound patient should have an assessment of tetanus immunization status. Facial lacerations, even if clean, are tetanus-prone injuries if they involve any contamination or devitalized tissue. Current guidelines recommend that if a child has not had a tetanus booster in the past five years (for dirty wounds) or 10 years (for clean minor wounds), a tetanus toxoid-containing vaccine (Tdap) should be administered. In practice, most children are up-to-date on their DTaP/Tdap series, but a booster may be indicated for older children or adolescents. Tetanus immune globulin (TIG) is reserved for children with uncertain or incomplete vaccination histories who present with high-risk wounds (deep punctures contaminated with soil, etc.) [25]. Fortunately, the incidence of tetanus from facial wounds is exceedingly rare.

Post-repair wound care

Managing pediatric facial lacerations involves much more than just closing the wound, as the care given in the days and weeks following the repair plays a pivotal role in the final healing process and the appearance of the scar. It is essential for both healthcare professionals and family members to work together during this recovery period. Providing families with clear, detailed instructions regarding wound care before leaving the clinic or hospital is crucial. Following these at-home care guidelines closely has been shown to contribute to better healing and fewer chances of complications [26].

Dressings and Moist Wound Care

Immediately after closure, most facial lacerations (if closed with sutures or strips) will be dressed with a light protective dressing. This could be a small piece of sterile gauze with tape or a commercial bandage. The primary purposes are to absorb any slight oozing, to protect the wound from dirt, and to prevent the child from touching it. For glue-repaired wounds, often, no dressing is applied (since the glue itself forms a protective film), but occasionally, a dry gauze is placed gently over the top if the child is likely to pick or if there is any bleeding. It is generally recommended to keep the initial dressing in place for about 24 hours, if possible, to avoid disturbing the wound during the very initial phase of healing. After 24-48 hours, caregivers can usually remove the bandage and begin daily gentle cleansing as instructed [27].

Notably, research has debunked the traditional belief that wounds must be kept dry for several days. Brief exposure to water, such as gentle washing or a short shower, after the first 24-48 hours does not increase the risk of infection [27]. In fact, gentle washing can help keep the wound area clean.

A critical concept in modern wound management is moist wound healing. A wealth of evidence shows that wounds allowed to heal in a moist environment re-epithelialize faster and with better quality than wounds that dry out and form a scab. In a moist environment, new skin cells (keratinocytes) can migrate over the wound more readily, and there is less intense inflammation, resulting in thinner, more supple scar tissue [28]. By contrast, a dry scab can act as a barrier to cell migration and can increase inflammation.

Therefore, we advise caregivers not to “air out” a fresh wound for prolonged periods, but rather to keep it lightly covered and hydrated. After the initial dressing is removed (at 24-48 hours), the caregiver should apply a thin layer of petroleum jelly (or antibiotic ointment if prescribed) to the wound at least twice daily and cover it with a fresh bandage. This keeps the wound bed moist and protected. If the child’s skin is sensitive to adhesive tapes, paper tape or a hypoallergenic dressing can be used.

For very small facial wounds, some clinicians may opt to leave them open after 24-48 hours but instruct the family to keep applying ointment frequently to prevent a hard scab from forming. The key is that a shiny coating of ointment should be maintained on the healing laceration so that if a scab forms, it is soft and minimal.

In practice, a good routine is to cleanse the wound once or twice a day with mild soap and water (no scrubbing, just letting water run over it or dabbing gently with a clean cloth), then pat the area dry, and immediately apply petroleum jelly. If the wound is in an area that can be covered (cheek, chin, or forehead), place a fresh bandage over it. If it is difficult to bandage (say, on the eyelid or near the lip), just be very diligent with keeping it moist with ointment and instruct the child (if old enough) not to pick at it. Parents should prevent the child from scratching or picking at the wound or any scab, as mechanical trauma is a common cause of worsened scarring. Covering the wound with a bandage can physically block picking. If itching is causing the child to scratch, an age-appropriate dose of antihistamine (such as cetirizine or diphenhydramine) could be given, or a silicone gel (discussed below) can also relieve itch when applied later.

Signs of Infection

Throughout the first two weeks, the wound is actively healing and is vulnerable to infection. Families should be counseled on signs of infection: increasing redness spreading from the wound, swelling, warmth, worsening pain, pus discharge, or fever. A slight rim of redness (erythema) around the wound and some swelling are normal in the first 48 hours, but this should improve, not worsen. If there is concern for infection, they must return for medical evaluation. Early infections can often be managed with oral antibiotics without disturbing the repair, but more severe infections might require suture removal and drainage. Fortunately, infections in well-managed facial lacerations are uncommon [29]. Emphasizing hygiene (hand-washing before touching the wound and keeping pets/dirt away from the wound) and adherence to the moist wound care regimen is typically a sufficient preventive measure.

Suture Management

If non-absorbable sutures were placed, caregivers should keep an eye on the wound for any loosening or redness along the stitch tracks. The wound should still be kept moist as above; applying ointment will also help prevent crusting on the sutures. Just before suture removal (around day 5), gently cleaning off any crust with dilute hydrogen peroxide or saline can facilitate easier removal. Early removal (day 3-5) followed by adhesive strips can be considered to minimize marks in very low-risk situations, but typically, day 5 is a safe balance for facial stitches [16]. If absorbable sutures were used, they usually start to fall out or dissolve by around one week [15]. Parents might see pieces of suture extruding; they should not pull at them forcefully, but they can trim any long ends with clean scissors. Often, by 10-14 days, most fast-absorbing sutures are gone. If any remain within two weeks, a clinician might elect to remove them to prevent them from causing irritation or trapping bacteria.

Following suture removal, application of adhesive strips across the wound for a few additional days can provide mechanical support, minimize wound tension, and further improve the final scar outcome [19].

Scar prevention strategies

After the initial wound healing phase, generally the first 1-2 weeks until the skin surface is closed, attention shifts to longer-term scar maturation. Over the weeks and months following a laceration, the scar undergoes remodeling as collagen fibers reorient and the scar often contracts and fades in color. This process typically continues for up to one year in most cases and may extend even longer in some scars. The proactive steps taken during this period can significantly influence the final cosmetic appearance. Evidence-based scar management typically begins about 2-3 weeks post-injury, once the new epithelium is intact and there are no open areas [30]. Early intervention is particularly important for scars that show signs of hypertrophy (raised, red, and thickened scars) or in patients with risk factors for hypertrophic scarring, such as a personal or family history of keloids or wounds that were under tension.

Silicone Therapy

Topical silicone is widely regarded as the first-line, gold standard preventive treatment for abnormal scars [10]. Silicone gel sheets have been used for decades in managing hypertrophic scars and keloids, and more recently, silicone gel ointments have become popular. A 2020 systematic review and meta-analysis of randomized trials confirmed that silicone gel significantly improves scar parameters, demonstrating reductions in scar pigmentation, height, and hardness compared to no treatment or placebo. In that analysis involving 375 patients, silicone-treated scars had notably better Vancouver Scar Scale scores in pigmentation and pliability domains than untreated scars. Importantly, studies have shown that silicone gel and silicone sheets are equally effective [11]. Although sheets may offer additional benefit by exerting some pressure and occlusion, gels are easier to apply to facial scars and are often better tolerated by children.

The exact mechanism by which silicone improves scars is not fully understood, but it is thought to act by hydrating the stratum corneum and modulating the microenvironment of the scar, possibly reducing collagen production and mast cell activity. Additionally, silicone may help regulate fibroblast activity to prevent overproduction of collagen. Given its efficacy and safety, silicone is recommended by international scar management guidelines for the prevention of hypertrophic scars [10].

For pediatric patients with facial scars, a silicone gel (topical) or thin silicone sheet can be started as soon as the wound is fully epithelialized (usually around two weeks after the laceration or 2-3 weeks post-repair). Using it too early (on an open wound) could risk maceration or infection, so one must ensure the skin is intact. Once begun, the silicone product should be applied or worn daily for at least 2-3 months for preventive benefit. Many experts advise continuing for six months or more if the scar remains active (red or raised) [3]. In practice, for a simple facial scar that is flat and pale by 8-12 weeks, silicone can probably be discontinued earlier, but for any scar that shows even mild hypertrophy, continuing up to six months is wise.

With children, compliance can be a challenge, so choosing the form of silicone that the child and caregivers find easiest is key. Silicone gel (e.g., in a tube that dries into a thin film) can be applied 1-2 times a day. It dries clear and is generally not noticeable, which is good for facial use. Older children can even apply it themselves like a “scar lotion” as part of their routine. Silicone sheets or patches can be cut to the size of the scar and placed over it, usually worn for 12 hours a day or more. For example, a small silicone sheet can be taped onto a child’s cheek scar each night and removed in the morning, achieving ~12 hours of wear per day. If using sheets on the face, one must secure them (with tape or an overlay bandage) and ensure the child will tolerate it (some very young kids may just peel it off).

Silicone sheeting has the advantage of providing mild pressure and not requiring reapplication throughout the day, but its adhesiveness might be an issue if a child’s skin is oily or moving a lot. Either way, multiple RCTs and meta-analyses support silicone use to prevent hypertrophic scars and to improve scar maturity [11]. For instance, O’Brien and Jones’s Cochrane review found that in patients at high risk of hypertrophy, silicone sheeting significantly reduced scar elevation and redness compared to no treatment [10]. We recommend that clinicians encourage the use of silicone therapy for any pediatric facial laceration that is more than trivial, as a prophylactic measure with a strong benefit-to-risk ratio.

Scar Massage

Once the initial tenderness of the wound subsides (usually by 2-3 weeks), gentle massage of the scar can be initiated. Scar massage involves rubbing and pressing on the scar and surrounding tissue in circular motions or along the orientation of the scar. The purported benefits of massage include softening the scar tissue, improving its elasticity, and possibly aiding in the alignment of collagen fibers during remodeling. Massage may also desensitize the area and improve pliability. A literature review on massage in scar management concluded that while high-level evidence was limited, many reports and smaller studies found improvements in scar softness and patient comfort with regular massage [30].

In pediatric burn patients, for example, massage therapy has been associated with reduced pruritus and improved scar texture [30]. Given that massage is low-cost or no-cost and can be easily taught to caregivers, it is a commonly recommended intervention despite the lack of large randomized controlled trials.

For pediatric facial scars, caregivers can be instructed to use a fingertip with a small amount of moisturizing cream or ointment (to reduce friction) and gently massage along the scar for a few minutes once or twice daily. Some protocols suggest massaging for 5-10 minutes at a time, but realistically, even a few minutes of attentive massage each day is beneficial. The pressure should be sufficient to blanch the scar slightly, but not so forceful as to cause pain or significant redness. Massage can be combined with the application of topical agents; for example, when applying silicone gel or vitamin E, caregivers can massage it gently into the scar.

Recommendations vary, but performing massage daily for at least six weeks is common, and continuing up to 3-6 months may yield additional benefits [30]. The evidence is “weak” regarding exact massage protocols, but given the potential benefits and minimal downsides, we advise incorporating scar massage into the care plan. Families should, of course, stop if the scar shows any irritation or if the child finds it too uncomfortable. In practice, many children enjoy or at least tolerate massage (some describe it as a mini “massage therapy” and get the child involved as a game or routine).

Sun Protection

New scars are highly vulnerable to ultraviolet (UV) radiation. UV exposure increases the risk of hyperpigmentation and worsens the appearance of immature scars. Pediatric patients have active melanocytes that respond robustly to UV radiation, making them particularly prone to post-inflammatory hyperpigmentation [3].

It is well documented that fresh scars younger than six months will darken disproportionately if exposed to sunlight or tanning beds [3]. Therefore, strict sun protection is universally recommended for at least 6-12 months post-injury. Caregivers should be instructed to apply a broad-spectrum sunscreen (SPF ≥ 30) daily once the wound is fully healed. Physical sunscreens containing zinc oxide or titanium dioxide are preferable, especially for young children. Sunscreen should be reapplied every two hours and after swimming or sweating. In addition, hats, shade, and protective clothing are encouraged as adjunctive strategies [3].

Even after one year, continued sun protection on scar sites is wise, as scars may remain more photosensitive than surrounding skin for extended periods. Consistent sun protection significantly improves long-term cosmetic outcomes by reducing scar redness and pigmentation [3].

Parental guidance and emotional support

In addition to physical wound care, providing emotional support is an essential component of the recovery process for children with facial lacerations. Caregivers should be encouraged to reassure the child in a calm, positive manner that the injury will heal and that any resulting scar will fade over time. Involving the child in simple aspects of wound care, such as assisting with ointment application under supervision, can foster a sense of control and reduce fear. Parents should avoid repeatedly drawing attention to the wound or expressing worry about its appearance, as this may increase the child’s anxiety. Instead, the healing process should be normalized through age-appropriate, reassuring conversations.

Caregivers should also be educated about the typical stages of healing, including initial redness, mild swelling, and gradual scar fading over several months, to help set realistic expectations. Awareness of emotional signs, such as social withdrawal or embarrassment, is important, and early referral to pediatric counseling services may be beneficial if needed. Empowering families with both practical wound care skills and emotional reassurance plays a critical role in promoting optimal scar outcomes and supporting the child’s psychological well-being during recovery [31].

Topical remedies (onion extract, vitamin E, etc.)

Many over-the-counter scar products contain ingredients such as onion extract (e.g., the popular product Mederma®), vitamin E, aloe vera, and other botanicals. It is important to counsel caregivers on what is proven and what is not, to set realistic expectations and avoid unnecessary expenses or skin reactions.

Onion Extract (Allium cepa)

Onion extract gel is one of the most marketed scar treatments. Earlier small studies suggested it might improve scar softness and redness, but more rigorous analysis has cast doubt on its effectiveness. A comprehensive meta-analysis published in 2021 evaluated onion extract products for scar management across 13 randomized controlled trials [12]. The findings were telling: onion extract gels showed no significant advantage over other topical treatments or even plain ointment in improving scar appearance [12]. Specifically, when comparing onion extract gel to petroleum jelly or silicone gel, there were no differences in scar outcomes as rated by investigators and patients [12].

Onion extract did show an improvement when compared to doing nothing at all, but any proactive treatment (even just massage with petroleum jelly) would likely do the same. Moreover, the meta-analysis found that onion extract gel was associated with a higher incidence of adverse effects such as skin irritation and rash. In fact, onion extract users were more likely to discontinue treatment due to intolerance (itching and erythema) than users of other products [12].

The authors concluded that onion extract gel has no special benefit in scar management and could even pose a higher risk of irritation. Interestingly, one subset of studies in that review indicated that onion extract combined with silicone (i.e., a silicone gel that also contains onion extract) performed better than treatments without those ingredients, suggesting any benefit is likely attributable to the silicone component [12].

In summary, current evidence does not support onion extract alone as an effective scar preventative, and it may cause dermatitis in some children. Clinicians can convey to parents that while products like Mederma® are popular, scientific data show they likely perform no better than plain moisturizers or silicone gel in improving scars [12]. If a caregiver is keen on using such a cream, it likely will not do harm if the child tolerates it, but they should monitor for any redness or allergy.

Vitamin E (Tocopherol)

Vitamin E oil is another commonly cited home remedy for scars. Many individuals break open vitamin E capsules and apply the oil directly to wounds in hopes of improving scar appearance. However, multiple studies have demonstrated little to no benefit of topical vitamin E alone on scar outcomes. A 2016 systematic review found that among six controlled studies, half showed no significant improvement in scar appearance with vitamin E use, and the remaining studies demonstrated only marginal benefits, often when vitamin E was used alongside other treatments [13].

Moreover, topical vitamin E was associated with contact dermatitis, including redness and itching, in a considerable number of patients. Approximately one-third of users in some studies developed dermatitis. The authors concluded that there is insufficient evidence to support the widespread use of topical vitamin E as a standalone scar treatment [13]. These findings align with previous studies showing no significant cosmetic benefit and potential harm through allergic reactions.

Thus, while keeping scars moisturized is beneficial, vitamin E itself does not appear to offer additional advantage beyond what any moisturizing agent would provide [13]. Parents should be advised that if they choose to use vitamin E, they should monitor for signs of irritation and discontinue use if a rash or other adverse reaction occurs.

Aloe Vera and Other Remedies

Aloe vera has some anti-inflammatory and wound-healing properties and is soothing, but like vitamin E, there is scant evidence that it makes a significant difference in scar outcomes beyond general hydration. Still, aloe is generally safe and can be used as a moisturizing agent during massage if desired. Honey, cocoa butter, shea butter, and other plant oils can similarly act as moisturizers but have not demonstrated superior scar prevention effects [28].

The bottom line with these remedies is that hydration is good. A well-moisturized scar will mature better than a dry one. The exact content of the moisturizer is less critical, as long as it does not irritate the skin [28].

Given the plethora of over-the-counter scar creams, a practical approach is to guide caregivers toward the proven interventions (silicone products, sun protection, and pressure/taping) as first-line, while clarifying that other creams likely have minimal impact beyond serving as moisturizers.

Pressure therapy and taping

In professional scar management, especially for burns or large surgical wounds, pressure garments are a known modality to prevent hypertrophic scar formation. On the face, however, pressure garments are impractical. A simpler analogue in small scars is the use of adhesive paper taping.

Recent evidence suggests that applying hypoallergenic paper tape (e.g., 3M Micropore®) over a healing scar for several weeks can significantly reduce scar hypertrophy by minimizing mechanical forces across the scar during its remodeling phase [14]. A 2021 comprehensive review found that non-stretch paper tape applied over linear surgical wounds led to narrower, flatter scars with less redness and itching compared to no taping [14].

Patients typically applied tape themselves and replaced it every 3-7 days. Over 12 weeks, studies showed that taped scars had superior cosmetic evaluations, and benefits persisted up to one year in some cases [14]. Taping is particularly helpful in areas subject to frequent movement, such as the chin or cheeks.

In practice, after suture removal or after the tissue adhesive has sloughed off, caregivers can be instructed to apply thin strips of paper tape along the scar. Taping should continue for 1-2 months, if possible, especially for scars on dynamic areas [14].

If used alongside silicone therapy, such as silicone gel during the day and tape reinforcement at night, these interventions can be complementary. Families should be advised to change the tape if it loosens or becomes dirty and to monitor the underlying skin for signs of irritation. Overall, adhesive taping is a simple, inexpensive, low-risk intervention that can meaningfully improve pediatric facial scar outcomes [14]. A summary of scar prevention interventions for pediatric facial lacerations is provided in Table 2.

**Table 2:** Scar Prevention Interventions for Pediatric Facial Lacerations RCT: randomized controlled trial, OTC: over the counter, PDL: pulsed dye laser

Intervention	Mechanism/benefit	Evidence and recommendations	Citations
Silicone gel or sheets	Occlusive silicone therapy hydrates scar and modulates collagen production, reducing hypertrophy.	Strong evidence from RCTs and meta-analyses shows significant improvement in scar height, color, and pliability. It is recommended to start approximately 2-3 weeks post-closure and continue daily for 2-6 months. Sheets and gels are equally effective; whichever the child tolerates may be used. Silicone therapy is considered the first-line scar prevention method for moderate- to high-risk scars.	O’Brien and Jones (2013) [10], Wang et al. (2020) [11], Barone et al. (2025) [3]
Sun protection (SPF 30+ sunscreen, hats)	Prevents UV-induced hyperpigmentation and long-term discoloration of scars.	Universally recommended for any new scar for at least 6-12 months. UV exposure causes new scars to darken and become more conspicuous. Strong consensus (level 5 evidence) for strict sun avoidance or protection.	Barone et al. (2025) [3]
Moist wound healing (petroleum ointment)	Maintains hydration in healing tissue, speeding epithelialization and reducing inflammation.	Extensive evidence that moist healing leads to faster re-epithelialization and less scarring. Randomized trials in acute wounds: petrolatum = antibiotic ointment in healing efficacy. The standard of care is to keep the wound moist until it heals.	Nuutila and Eriksson (2021) [28], Draelos et al. (2011) [18]
Scar massage (after ~2-3 weeks)	Mechanically softens scar tissue, may promote collagen remodeling and improve pliability; also helps desensitize.	Some clinical studies and reviews support improved scar softness and patient comfort. Limited high-level RCT data, but widely practiced (level C evidence, recommended by consensus). Perform daily for at least 6 weeks.	Shin and Bordeaux (2012) [30]
Adhesive paper tape (micropore)	Offloads tension across scar, supports it during remodeling, and provides occlusion.	RCTs and systematic reviews show significantly reduced scar width and hypertrophy with tape used for 12 weeks post-closure; especially useful on high-movement areas (chin and cheeks).	Atkinson et al. (2005) [14]
Onion extract gel (e.g., Mederma®)	Claimed anti-inflammatory and fibrinolytic properties; popular OTC scar gel.	No proven benefit over other treatments. A 2021 meta-analysis found that onion extract was not superior to petrolatum or silicone. Increased risk of localized irritation. Not recommended as a standalone therapy.	Yuan et al. (2021) [12]
Vitamin E oil	Antioxidant; traditionally thought to help scars.	Controlled trials do not demonstrate significant improvement. Some cases of contact dermatitis (up to 33%). Insufficient evidence for benefit. If used, monitor for rash.	Tanaydin et al. (2016) [13]
Pressure devices (garments or custom pads)	Constant pressure flattens scars and reduces blood flow, deterring hypertrophic scar growth.	Typically used for large burn scars. Not practical for small facial scars, except possibly using tape. High-level evidence in burns.	Zurada et al. (2006) [19]
Laser therapy (PDL, fractional laser)	Improves scar color (PDL for redness) and texture (fractional for remodeling).	Not OTC. PDL can significantly reduce redness and thickness in hypertrophic scars. Consider if the scar remains problematic after 1-2 months.	Barone et al. (2025) [3]
Intralesional corticosteroid	Reduces excessive collagen (flattens hypertrophic scars/keloids).	Strong evidence in hypertrophic scars and keloids. Not used prophylactically in new traumatic scars unless early hypertrophy is observed.	Zurada et al. (2006) [19]

Limitations

This review is limited by the availability of high-quality randomized controlled trials specifically focused on pediatric facial lacerations and scar prevention. While many recommendations are supported by adult data or expert consensus, pediatric-specific evidence remains relatively limited in certain areas, such as the use of pressure taping and scar massage. In addition, individual healing responses vary based on genetic factors, wound characteristics, and adherence to post-repair care, which can influence cosmetic outcomes despite optimal management strategies. Future pediatric-specific prospective studies are needed to strengthen the evidence base for scar prevention interventions.

## Conclusions

Facial lacerations in children, while initially alarming, often heal very well with rapid and evidence-based management. Essential strategies include meticulous wound cleansing, the use of child-appropriate closure techniques such as topical LET gel and tissue adhesives, and limiting antibiotics to only those cases where infection risk is present. Moist wound care and personalized closure choices support optimal healing during the acute phase and lay the foundation for favorable outcomes.

Early attention to scar prevention is crucial, relying on interventions such as silicone gels or sheets, gentle massage, sun protection, and tension offloading with paper tape. When caregivers receive clear education and participate actively in daily wound care, and when providers implement proven, up-to-date practices, children usually experience excellent healing with minimal visible scarring. This comprehensive and collaborative approach not only ensures physical recovery but also contributes positively to the child’s emotional well-being.
